# Impact of infection control activities on the rate of needle stick injuries at a tertiary care hospital of Pakistan over a period of six years: an observational study

**DOI:** 10.1186/1471-2334-9-78

**Published:** 2009-05-29

**Authors:** Afia Zafar, Faiza Habib, Roshan Hadwani, Muslima Ejaz, Khurshid Khowaja, Rozina Khowaja, Seema Irfan

**Affiliations:** 1 Department of Pathology and Microbiology, Aga Khan University Hospital, Karachi, Pakistan; 2 Divisions of Nursing, Aga Khan University Hospital, Karachi, Pakistan

## Abstract

**Background:**

Accidental exposure to blood and body fluids is frequent among health care workers. They are at high risk of nosocomial transmission of blood borne pathogens due to injuries caused by used sharps. We are reporting impact of surveillance and educational program on the rate of needle stick injuries among health care workers at a tertiary care hospital in Pakistan.

**Methods:**

At Aga Khan University Hospital sharp injuries are reported to infection control office. To reduce these incidents a quality improvement project was inducted in the year 2005. Health care workers were educated; surveillance data from 2002 to 2007 was analyzed and compared with various risk factors.

**Results:**

During study period 1382 incidents were reported. Junior doctors sustained highest number of injuries (n = 394; 28.5%) followed by registered nurses (n = 283; 20.4%). Highest number of incidents was reported during blood collection (19%). An increasing trend was observed in the pre intervention years (2002–04). However noticeable fall was noted in the post intervention period that is in year 2006 and 2007. Major decline was noted among nurses (from 13 to 5 NSI/100 FTE/year). By relating and comparing the rates with various activities directly linked with the use of syringes a significant reduction in incidents were found including; hospital admissions (p-value 0.01), surgeries and procedures performed (p = 0.01), specimens collected in the laboratory (p = 0.001) and patients visits in clinics (p = 0.01).

**Conclusion:**

We report significant reduction in needle stick injuries especially during post intervention study period. This is being achieved by constant emphasis on improving awareness by regular educational sessions, implemented as a quality improvement project.

## Background

Healthcare workers (HCWs) are at direct risk of exposure to blood and other body fluids during the course of their job. Consequently, they are at risk of infection of blood borne viruses including hepatitis B virus (HBV), hepatitis C virus (HCV) and human immunodeficiency virus (HIV) [1]. Occupational exposure to blood can result from percutaneous (needle stick or other sharps injury) and mucocutaneous injury (splash of blood or other body fluids into the eyes, nose or mouth), or blood contact with non-intact skin [2]. Needle stick injury (NSI) is the most common form of occupational exposure to blood which results in transmission of blood borne infections.

Numerous modifiable and non-modifiable factors place HCWs at risk of NSIs. The most common reasons are two-handed recapping, and the unsafe collection and disposal of sharps waste. Personnel in areas such as operating, delivery and emergency rooms and laboratories have higher risk of exposure [3]. Cleaners, waste collectors and other employees involved in handling blood-contaminated items are also at risk.

Globally in the year 2000 approximately 16,000 HCV, 66,000 HBV, and 1,000 HIV infections occurred in HCWs due to percutaneous injuries [4]. HCV, HBV, and HIV in HCWs attributable to percutaneous occupational exposure were 39%, 37%, and 4.4% respectively [5]. Centers for Disease Control and Prevention (CDC) estimates that approximately 385,000 needles and sharps-related injuries occur every year to HCWs in the United States [6].

Biohazard exposures are often accepted as being a part of the job although majority of these exposures are preventable. In the United States, NSIs decreased from an estimated one million exposures per year in 1996 to 385,000 per year in 2000. This decline resulted from continuous surveillance and implementation of preventive measures.

In contrast, in resource limited countries like Pakistan, biohazard exposures and their consequent health impacts are rarely monitored. However higher rates of hepatitis B and C infections have been reported from Pakistan [7,8]. These produce an immense burden on an already compromised health system; hence accurate information regarding the magnitude of NSI in our population is crucial to be able to undertake prevention efforts that would have an overall impact on the quality of health care. Thus, we are reporting for the first time in Pakistan surveillance data of NSIs in HCWs at a tertiary care hospital. Different studies have reported a decline in NSI rate after implementation of multifactorial approaches including safety engineered devices and reusable sharps containment system [9]. Being a developing country, financial constrains do not allow us to utilize these sophisticated preventive measures. Health care workers education is essential for every intervention and can be effective on its own [10]. To achieve this, we designed a strong NSI prevention educational program and implemented this as a quality improvement project during year 2005 to reduce the rate of NSIs in HCWs at a tertiary care hospital. We report the strong effect of NSI prevention educational program on the rate of NSI in HCWs at a tertiary care hospital.

## Methods

Aga Khan University Hospital (AKUH) is a 550-beded tertiary care referral teaching hospital located in Karachi, Pakistan. In this observational study, data of NSIs reported to the infection control office (ICO) from January 2002 to December 2007 was analyzed. Incidents were included if the event involved a percutaneous exposure with a contaminated device. All clean NSIs (injuries due to unused sharps) were excluded as they pose almost no risk of transmission.

As per hospital policy, all incidents of NSIs as well as blood and body fluid exposures are reportable to ICO within 24 hours of occurrence. On routine basis, following an event HCW reports the incident to IC nurse (ICN). Subsequent to that, ICN collects the relevant information including time of incident, its reporting time, nature of incident, its association with the activity involved such as surgery, recapping, handling of intravenous line, safety of sharp disposal, compliance of standard precautions, HBV immunization status of recipient and HBsAb level. In cases where source is known, their antibody against HCV and HIV are checked to eliminate the chances of disease transmission. In cases of unknown donor or source, we follow the case as per CDC guidelines. All information is entered into computerized standardized forms devised to maintain records, and for follow-up of individual cases.

To reduce the number of NSI a quality improvement project was inducted in year 2005. To assess the impact of educational program, we analyzed the data by dividing the study period into three groups as pre-intervention (2002 to 2004), intervention (2005) and post intervention periods (2006 to 2007). Comparisons were made on mean values for multiple variables which included: job classification, clinical department, when the injury occurred in relation to the use of the device, number of staff, procedures performed, patient visits in clinics and their admission and samples collected in the laboratory over the years.

During pre-interventional period (2002 to 2004) all new employees were supposed to attend an educational session conducted by ICO. For onboard staff, scheduled sessions were conducted on a regular basis as shown in table 1. In year 2005 a quality improvement project was introduced to reduce the number of NSIs. During this period, a multifaceted educational approach was adopted to reinforce safe practices. That included more educational sessions for both new and on board employee as well as introduction of interactive sessions for support services staff. This group was not targeted in the past. Table 1. In the beginning of 2005 an IC certification course of one week duration was organized for 46 HCWs, serving as in-charge positions in various clinical areas. They were mainly chosen from the nursing division but a few paramedics from support services and senior residents were also included. Many of these HCWs were then nominated as infection control monitors in their respective units. Monthly meetings were regularly conducted to get feedback and to resolve on going issues as shown in table 1. In addition, infection control week was celebrated in 2005 and 2006, during which lectures were delivered by members of the infection control committee; flyer and poster competitions were arranged; IC message of the month was disseminated by electronic mails across the hospital. Workshops were conducted on standard precautions such as seven steps of hand hygiene, and appropriate use of personal protective equipments, and safe and immediate disposal of sharps was ensured in areas where appropriate disposal was an issue.

**Table 1:** Infection control educational sessions conducted in the year 2004 & 2005

	Number of sessions	
	
Educational sessions	2004	2005
Nursing staff	2	6

ICU residents & faculty	2	12

Sessions for postgraduates	0	6

Training & development of new employees at the time of induction	12	32

Sessions for onboard staff	35	37

Meeting with Infection control monitors	0	12

In individual units	3	22

Laboratory staff	2	10

House keeping & ward-aid	1	7

Laundry, CSSD & distribution	0	5

Food & Nutrition	0	4

Others*	0	9

* Physiotherapy, cardiopulmonary, neurophysiology & maintenance department

In year 2005, approximately 40% of doctors, 90% of nursing, 95% of domestic and 50% to 80% of supportive care staff were educated or trained for standard precautions including preventive measures for NSIs.

Frequencies, rates and proportions were calculated using SPSS for windows and statistical significance was observed using Chi-square test [11]. Annual rates of NSIs were calculated using the number of HCW positions for each year as denominator. Determination of the confidence intervals for the rates of NSIs among HCWs per year for the entire study period was performed using the guidelines of Gardner and Altman in order to quantify any statistical uncertainty [12]. For comparison of proportion, a p-value of < 0.05 was considered statistically significant.

## Results

Through self-reporting surveillance system, overall 1382 NSIs were reported by the HCWs from January 2002 to December 2007. An increasing number of NSIs were noticed from 2002 to 2005. However, in the year 2006 and 2007, incidents were reduced. Table 2. When we compared rates between NSI during pre-intervention and post-intervention periods, we found a statistically significant decrease in number of NSIs (p = 0.03). NSI was calculated per 100 full time doctors and nurses per year as they sustained majority (50%) of injuries. It was found that incidents were reduced in both groups but major decline was noted among nurses that are from 13 to 5 NSI/100 FTE/year. Table 3.

**Table 2:** Needle stick injuries among health care workers from 2002–2007

Years	# NSI/HCWs	Rate (95% CI*)
**2002**	208/4788	4.3 (3.7 – 4.8)

**2003**	246/5261	4.6 (4.04 – 5.1)
**2004**	255/5861	4.3 (3.7 – 4.8)

**2005**	278/6566	4.2 (3.7 – 4.8)

**2006**	201/7635	2.6 (2.2 – 2.9)

**2007**	194/7468	2.5(2.1 – 2.8)

* Confidence Interval

**Table 3:** Number of NSI per 100 full-time doctor & nurses per year

Year	Doctors			Nurses		
	
	# of doctors	# of NSI	NSI/100 FT doctors/year	# of nurses	# of NSI	NSI/100 FT nurses/year
**2002**	310	51	17	460	59	13

**2003**	323	86	27	490	43	9

**2004**	329	71	22	504	44	9

**2005**	374	74	14	520	57	11

**2006**	414	51	12	585	50	9

**2007**	437	61	14	611	30	5

With the assistance of IT and human resource departments, computerized data of various indicators directly related to use of sharps were gathered. Analysis revealed that on average, growth for in-patients was 4.8% and out patient services was 10%. By relating and comparing the rates of years 2002 and 2006 with various activities directly linked with NSIs such as hospital admissions (p = 0.01), procedures performed (p = 0.01), specimens collected (p = 0.001) and patients visits in clinics (p = 0.01), an overall reduction was found. Table 4.

**Table 4:** Growth of health care workers and various indicators

Years	Number of HCWs	Hospital admissions	Surgeries performed	Specimens collected	Patients visits in clinics
**2002**	4788	33,565	7800	1,093,856	364,354

**2003**	5261	34,440	9536	1,259,888	383,981

**2004**	5861	35,670	8364	1,325,178	425,000

**2005**	6566	38,612	8931	1,578,796	498,000

**2006**	7635	40,673*	9271	1,796,931*	537,000*

* 'p'-value ≤ 0.05 was considered significant in the growth of respective categories

**Figure 1:**
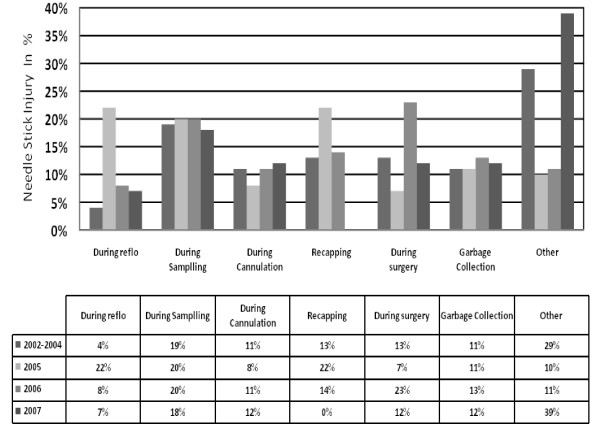
**Proportions of Needle Stick Injuries among health care workers from 2002–2007**.

Junior doctors (interns and residents) sustained the highest number of injuries (n = 394; 28.5%) followed by registered nurses (n = 283; 20.4%). Table 5. Further analysis of activities leading to NSIs revealed that throughout the study period, highest number of incidents were reported during blood collection (19%) followed by recapping, during clamping technique for blood sugar check by glucometer, while performing surgeries, waste disposal, and then cannulation. Figure 1. Around one fourth of the incidents occurred after use of sharps, which included recapping and clinical waste disposal. In post-intervention period, number of NSI due to recapping reduced to zero in year 2007. All NSIs among domestic staff occurred while collecting clinical waste or during environmental cleaning, and the percentage remained the same in pre and post intervention periods.

**Table 5:** Needle stick injuries among various categories of health care workers from 2002–2007

Years	Doctors	Nurses	Domestic	Phlebotomists	Technicians	Midwives	Others*	# of NSI
**2002**	51	59	26	14	28	6	24	208

**2003**	86	43	22	20	29	3	43	246

**2004**	71	44	39	36	15	3	47	255

**2005**	74	57	39	23	27	2	56	278

**2006**	51	50	23	17	16	2	42	201

**2007**	61	30	18	11	42	4	28	194

**Total**	394	283	167	121	157	20	240	1382

**%**	28.5	20.4	12	8.7	11.5	1.4	17.5	100

* Students, CSSD, kitchen, laundry and security staff

## Discussion

This study shows significant fall in the incidents of NSI. Apparent number of NSIs remained unchanged from 2002 to 2005. However, a sharp decline in the rate was noticed in the year 2006–07. Initially, it was presumed that maybe strategies adopted by infection control committee improved awareness and therefore the reporting. However, by relating and comparing the rates of years 2002 and 2006 with various activities directly linked with NSIs such as hospital admissions, surgeries and procedures performed, specimens collected in the laboratory and patients visit in clinics, an overall statistically significant reduction in reported NSIs was found. It appears that it is the outcome of constant emphasis on reporting of such injuries, frequent educational sessions and especially the quality improvement project which was initiated in the year 2005 by the infection control committee, aimed to reduce the incidents in 2006 and 2007.

In this study, the average rate of NSIs among HCWs per year is 3.76/100 in full time employees (FTE). A similar Australian study reported a rate of 9.4/100 FTE per year in nursing staff. Another study reported hospital mean rate of 8.79/100 FTE per year over 10-year survey period [13,14]. One study is comparable, as it too followed NSI rates in a single institute after multifaceted interventions. The authors assessed NSI rates per year over an eight-year period (1990–1998) [15]. In our report, low rate of NSIs could be explained by underreporting from all categories of staff, especially senior doctors, as during the entire study period only one senior surgeon reported the incident. Despite the fact that they experience most exposure and injuries due to nature of their job, they are known for underreporting [16-19].

Through out the study period, junior doctors reported the highest number of incidents. Compliance to universal precaution, improper handling of sharps and negligence towards safe practices are important issues we face at our institute. This finding is similar with a study published from a tertiary care university hospital based in London. Authors reported that majority of junior doctors were not following universal precaution during their day-to-day work [20]. Each year around a hundred and fifty new interns and residents join this university hospital for training. They graduate from other medical colleges where by large infection control activities do not exist. Prior to induction they go through a short orientation program, which includes a brief session on infection control. Besides that, amongst junior doctors work-stress and fatigue-related clinical errors are also known [21,22]. Present data clearly indicates that supervised training, especially during initial stressful years, is needed not only to reduce the incidents of NSIs but also to improve work performance [23,24].

In our institute waste segregation is performed at the site of its generation in designated color-coded boxes or bags and is then sent for incineration. On average 12% of incidents were reported by domestic staff. Almost all injuries to this group were associated with cleaning of environment and inappropriately disposed used sharps during waste disposal. They remain at risk of blood and body fluid exposures, mainly due to careless attitude and inappropriate waste disposal of used needles by medical and paramedical staff. Recapping was discouraged specifically in every educational session conducted by infection control group. May be this is the reason which improved the practice among HCWs.

Due to cost constraints, use of syringes with safety devices could not be introduced. This data shows that exposure of sharps after their use (recapping and waste disposal) was around 24–33%. It seems that further reduction in NSI is possible, provided we introduce engineered items with safety devices. Their use in the developed world has reduced the number of NSIs [23-25]. Unfortunately, these devices are considerably much more expensive than the usual syringes. Their substitution carries noticeable economical impact directly on patients in the private sector and to national health programs in the public sector in a resource limited country. However, the benefits and limitations of such intervention need careful assessment against the background epidemiology of hepatitis B, C and HIV as well as of percutaneous exposures within a given health care setting and in the country [13].

Estimated prevalence of Hepatitis B in Pakistani population is 3–4% and Hepatitis C is 6% [7,8]. A previous study conducted by Mujeeb et al at a public tertiary care hospital, Karachi among operating room personnel reported higher rates of NSIs and prevalence of Hepatitis B and C [26]. Another paper published in 2002 from AKUH Karachi reported 4% transmission of hepatitis C among HCWs after having NSIs [27]. These findings strongly suggest that to minimize occupational exposures and their devastating consequences the introduction and use of devices with safety measures is pertinent in this part of the world.

From previous studies it is evident that education, on going quality improvement projects and preventive programs play a major role in augmentation of knowledge and safe behavior of HCWs [10,13,26-30]. Likewise, in our study, decline in the rate of reporting followed significant fall in 2006 and in 2007. It seems that it is the direct outcome of initiation of institutional NSI quality improvement project, vigorous education and regular audits carried out prior to JCIA accreditation. Moreover, this is in concordance with the study published by Williams et al [31]. He reported that HCWs with more training in universal precaution were less likely to recap needles and more likely to wear gloves.

The strength of this study is that it is the first report from Pakistan showing reduction in NSIs due to organized and consistent effort of the infection control team of a private teaching tertiary care hospital. The study also has limitations: firstly, the data of NSIs was analyzed retrospectively, therefore in a few cases, entries were found incomplete, and as a result categorization of incident was found difficult, hence we could not run multivariate analysis for the risk factors association. Secondly, it is only one hospital data and does not represent other local institutes. Thirdly, under reporting from staff especially senior clinicians is a concern though all expenses of incident and follow up was borne by the hospital management.

## Conclusion

In conclusion, ongoing surveillance, organized structured training on occupational exposure to blood body fluid prevention and universal precautions improved the knowledge and behavior, as well as reduced the incidents amongst HCWs. This study also supports the idea of introducing basic infection control to the curriculum of all medical schools and nursing colleges as well as to other paramedical and technical staff training institutes.

## Competing interests

The authors declare that they have no competing interests.

## Authors' contributions

AZ conceived, designed and supervised the study. RH and RK collected the data. KK, RH and RK participated in implementation of the study. AZ, FH, SI and ME contributed in manuscript writing. FH and ME analyzed the data statistically. All the authors have read and approved the final manuscript.

## Pre-publication history

The pre-publication history for this paper can be accessed here:

http://www.biomedcentral.com/1471-2334/9/78/prepub
